# Diet influences the bacterial and free fatty acid profiles of the cuticle of *Galleria mellonella* larvae

**DOI:** 10.1371/journal.pone.0211697

**Published:** 2019-02-07

**Authors:** Michalina Kazek, Agata Kaczmarek, Anna Katarzyna Wrońska, Mieczysława Irena Boguś

**Affiliations:** 1 The Witold Stefański Institute of Parasitology Polish Academy of Sciences, Twarda, Poland; 2 BIOMIBO, Warsaw, Poland; Universidade de Sao Paulo Faculdade de Filosofia Ciencias e Letras de Ribeirao Preto, BRAZIL

## Abstract

The evolutionary success of insects is arguably due to their ability to build up a complex, highly-adaptable and very effective defense system against numerous pathogens, including entomopathogenic fungi. This system relies on the humoral immune system and cellular defense reactions. The first line of defense against biological pathogens is a cuticle formed of several layers. The cuticular lipids may contain hydrocarbons, free fatty acids (FFA), alcohols, waxes, glycerides, aldehydes and sterols. Cuticular fatty acids may also play a role in defending against fungal invasion. Our present findings show that the diet of insects can have a significant effect on their sensitivity and defense response to pathogens; for example, while *G*. *mellonella* larvae fed on beeswax had a similar appearance to those reared on a semi-artificial diet, they possessed a different cuticular free fatty acid (FFA) profile to those fed on a semi-artificial diet, and were less sensitive to *Conidiobolus coronatus* infection. It is possible that the presence of heneicosenoic acid (C21:1) and other long-chain free fatty acids (C22:0, C24:0, C26:0), as well as *Brevibacillus laterosporus* bacteria, on the cuticle of larvae fed on beeswax, plays a protective role against fungal invasion. Insect pests represent a global problem. An understanding of the basic mechanisms underlying the fungal infection of insects might provide a clearer insight into their defenses, thus allowing the design of more effective, and environmentally-friendly, means of controlling them. The greater wax moth is an excellent model for the study of immunology resistance. Knowledge of the influence of diet on pathogen resistance in insects can be also useful for creating a model of human diseases caused by pathogens, such as *Candia albicans*.

## Introduction

Insects comprise the most numerous and widespread class of animals. Their importance to life on earth is huge: they play an important role in the circulation and distribution of organic matter, participate in plant reproduction by pollination and form part of the diet of many vertebrate animals. However, despite the benefits some species bestow, many others are considered pests or vectors of various diseases [1–3].

Insects achieved evolutionary success thanks to the development of a complex, highly-adaptable and highly-effective defense system against numerous pathogens, including entomopathogenic fungi.The first line of defense against biological pathogens or insecticides is a cuticle formed of several layers, comprising an epicuticle on the outside, a procuticle underneath and an epidermis beneath that. The epicuticular layer is also covered by wax, which plays an important role as a barrier against water loss [4, 5]. About 70% of the cuticle is typically composed of proteins, with the remaining 30% being made up of lipids, chitin, chinons and phenols. The insect cuticle is also covered with complex mixtures of mainly nonpolar and polar compounds [6, 7]. It is possible that specific cuticular lipids play a role in prey recognition by specialized and predatory insects. The cuticular lipids may contain hydrocarbons, free fatty acids (FFA), alcohols, waxes, glycerides, aldehydes and sterols. The FFA profile can differ between insect groups, and sometimes within the developmental stages of the same species, and some cuticular lipids may have a role in defending against fungal invasion [6, 8]. The presence of antagonistic microbes, toxic lipids and cuticle phenolic compounds has been suggested to inhibit fungal growth [9, 10]. Cuticular fatty acids are also known to have a profound effect on fungal spore germination. For example, the remarkable resistance to mycosis of *C*. *vicina* larvae can be attributed to the composition of fatty acids present in its epicuticle [6, 11, 12].

The greater wax moth, *Galleria mellonella* (Lepidoptera order) is a member of the Galleriinae subfamily within the family Pyralidae[13]. It is an omnipresent pest of two species of honeybee: *Apis mellifera* and *Apis cerana*, particularly in the tropical and sub-tropical regions. The moth first enters their hives, whereupon its larvae dig into the cells containing pollen, the bee brood and honey, resulting in severe damage to the hive and the bee population; in fact, it is believed that the greater wax moth is one of the contributing factors to the decline of domestic and wild honeybee populations.

However, *G*. *mellonella* larvae have recently been used as an alternative to vertebrates as model hosts for studying pathogenic microorganisms [14–16]. The larvae offer many advantages as a model host. Most importantly, they can be maintained at 37°C, thus allowing microorganisms to be studied under the temperature conditions at which they are pathogenic to human hosts. In addition, they allow multiple options to be used for facile delivery of the pathogen: injection, oral delivery or topical application. *G*. *mellonella* larvae are also cheap and easy to maintain. Furthermore, this species is known to be susceptible to 29 species of fungi, seven viruses, one species of parasite and 16 biological toxins [16].

The main aim of this study was to determine whether the susceptibility to fungal infection demonstrated by two populations of greater wax moth (*G*. *mellonella*) larva, i.e. one maintained on a natural diet and the other fed a semi-artificial diet, is correlated with their cuticular FFA profiles, as determined by GC/MS technique. The study also examines the bacterial microflora on the cuticle of both *G*. *mellonella* populations. A significant novel element of our research is that this is the first examination of the influence of diet on the resistance of *G*. *mellonella* to entomopathogenic fungi. This study not only provides important insights into the field of insect physiology and insect immunology but also represents a useful reference for future studies.

## Materials and methods

### The fungus *Conidiobolus coronatus*

*C*. *coronatus* (Entomopthorales), isolate number 3491, originally isolated from *Dendrolaelaps* spp., was received from the collection of Prof. Bałazy (Polish Academy of Sciences, Research Center for Agricultural and Forest Environment, Poznań).

The fungal colonies were routinely cultured in 90 mm Petri dishes on Sabouraud agar medium. They were incubated at 20˚C under a 12-hour photoperiod (L:D 12:12) to stimulate sporulation [17]. Homogenized *G*. *mellonella* larvae were added to the medium to a final concentration of 10% wet weight (SAB-GM) in order to elevate virulence. The fungal colonies used for the experiments were cultured for seven days.

### Insects

Two cultures of wax moth, *G*. *mellonella* (Pyralidae, Lepidoptera), were reared in glass chambers at 30°C, 70% relative humidity and in constant darkness. One group was maintained on a semi-artificial diet composed of wheat flour, wheat bran, dry milk, corn flour, dry yeast, glycerine, honey and water, as described by Sehnal [18] The insects from the second group were reared under the same optimal growing conditions, but were fed with natural beeswax taken from natural hives.In each group, at least five generations were bred under these conditions. Upon reaching their final (seventh) instar, having ceased feeding before entering metamorphosis (five-day-old last instar larvae, 5DL7), the larvae were weighed and used in the experiments.

The naturally and semi-artificially fed 5DL7 larvae were exposed for 24 hours to fully-grown and sporulating *C*. *coronatus* colonies. Around 15 individuals were maintained in each Petri dish, and a control group was formed of larvae exposed for 24 hours to sterile SAB-GM. This was found to be the most efficient method of infection, being one that most closely resembles the natural infection process [19]. After exposure, the insects were transferred to new, clean Petri dishes with appropriate food and their further development was inspected daily. The insects intended for cuticular lipid extractions were frozen and kept at -80°C until analysis.

### Identification of bacteria

To identify the bacteria present on the *G*. *mellonella* cuticle, five larvae and 100 ml of sterile phosphate-buffered saline (PBS) were collected to a 250 ml sterile flask and shaken for 10 minutes. After this time, 500 μl of the suspension was taken and cultured on Petri dishes with Columbia Agar supplemented with 5% sheep blood. The colonies were incubated for 24 hours at 30°C (optimal growing conditions for *G*. *mellonella* larvae). For each culture, five independent repetitions were performed. In the next step, single bacterial colonies were isolated and cultured on new Petri dishes with Columbia Agar with 5% sheep blood. Again, the dishes were incubated for 24 hours at 30°C. For preliminary selection, Gram staining was performed. Microscope examination was performed using an Axio Vert A1 (Zeiss) microscope, and image acquisition with Zen (Zeiss) software.

The bacteria present on the insect cuticle were identified using a Vitek 2 Compact system (Biomerieux). VITEK GP cards for Gram-positive bacteria and VITEK BCL cards for Bacillus strains were used after microscopic identification. In the assays, 0.5–0.6 McFarland bacterial suspensions were used for the GP card and 1.8–2.2 McFarland suspensions for the BCL card.

The Minimum Inhibitory Concentration (MIC) values of gentamicin, vancomycin, chloramphenicol and tetracycline against the isolated bacteria were determined using E-test (Biomerieux). Bacterial suspensions (density 0.5 McFarland) were surface-cultured on Petri dishes with Mueller Hinton Agar medium (Biocorp). Following this, E-test bars were put on the dishes, and the MIC values were read after a 24-hour incubation period at 37°C. For each isolate, three independent replications were performed.

### Extraction of cuticular free fatty acids (FFAs)

To isolate the surface lipid components for GC/MS analysis, the larvae were extracted for five minutes in 20 mL of dichloromethane (Sigma). The extracts were then placed in glass flasks and evaporated under nitrogen. Table 1 includes the numbers of insects used, as well as the masses of the extracts.

**Table 1:** The occurrence of bacterial species on the cuticle of *Galleria mellonella* larvae reared on natural and semi-artificial food.

Bacterial species	Natural diet		Semi-artificial diet	
	N	Extensity [%]	N	Extensity [%]
***Bacillus cereus***	25	ND	25	32 ± 2.3
***Bacillus subtilis***	25	15 ± 2.7	25	23 ± 3.2
***Brevibacillus laterosporus***	25	68 ± 4.4	25	ND
***Enterococcus casseliflavus***	25	36 ± 6.2	25	42 ± 4.6
***Enterococcus faecalis***	25	ND	25	56 ± 3.8
***Kocuria kristinae***	25	ND	25	12 ± 5.1

N—number of insects; ND—not detected

Extensity—percentage of isolates in which a given bacteria species was identified.

### Derivatization method

Trimethylsilyl esters (TMS) of FFAs were obtained by the addition of 100 μl of BSTFA:TMCS mixture 99:1 (Sigma) to 1 mg of each extract, and heating for one hour at 100°C. The TMSs of the fatty acids were then analyzed by GC/MS.

### GC/MS analyses

The analyses were carried out with a GCMS-QP2010 system with mass detector (Shimadzu) and NIST 11 mass spectra database. Helium was used as the carrier gas. The injection mode was split. A ZB-5MSi (Zebron) column was used (thickness 0.25 μm, length 60m, diameter 0.25 μm) The column oven temperature cycle was held at 80 ^o^C for three minutes and then ramped from 80 ^o^C to 310 ^o^C at 4 ^o^C/minute; the final temperature was then held for 10 minutes. The ion source temperature was 200 ^o^C. The interface temperature was 310 ^o^C.

All compounds were identified on the basis of fragmentation patterns and on the basis of silyl derivative ions and the NIST 11 library. The mass spectrum of trimethylsilyl esters of fatty acids revealed the presence of the following ions: M+ (molecular ion), [M-15]+, and fragment ions at m/z 117, 129, 132 and 145. The 19-methylarachidic acid (Sigma-Aldrich) (1 mg/ml) was used as an internal standard (IS). The contents were calculated from the relative peak areas that were compared to the IS peak area and expressed as the percentage (%, w/w) of total extracts. Response factors of one were assumed for all constituents.

### Statistics

The obtained results were tested using the nonparametric Student’s t-test and one-way ANOVA, with the results being significant at p≤0.05. STATISTICA software (StatSoft Polska) was used for the evaluation of data. The obtained p-values are presented in the Results section.

## Results

### The variations in the sensitivity of *G*. *mellonella* larvae reared on natural and semi-artificial diet to fungal infection

**Fig 1:**
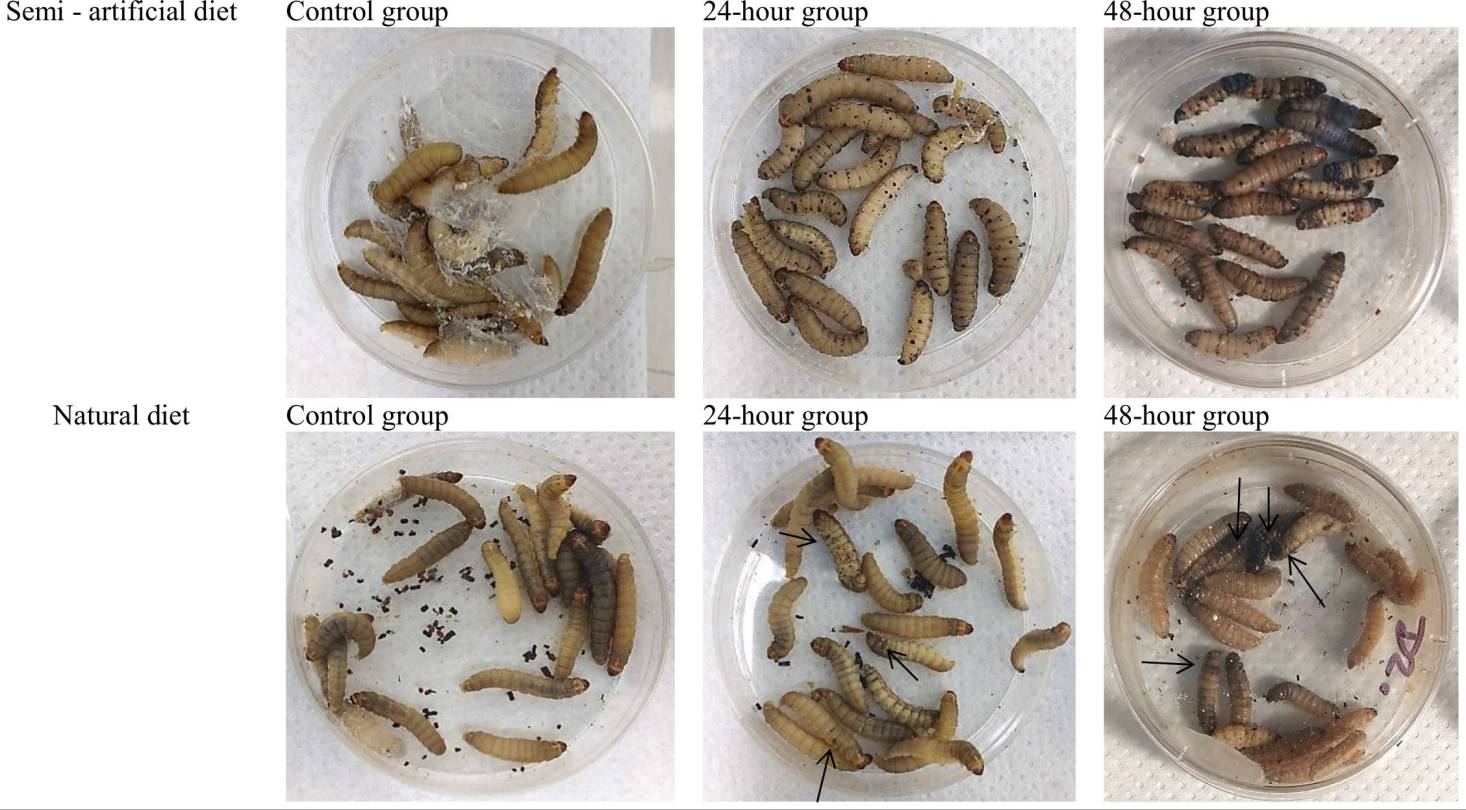
Effects of fungal infection on *G*. *mellonella* larvae maintained on semi-artificial (A) and natural (B) diets. Black spots on the larval bodies indicate changes occurring during fungal infection.

The exposure of *G*. *mellonella* larvae cultivated on semi-artificial diet to sporulating colonies of *C*. *coronatus* resulted in the appearance of black spots on the cuticles of all insects (N = 80) at the termination of exposure (Fig 1). The mortality of larvae after contact with the fungal pathogen was 32.5% after 24 hours and 100% after 48 hours. In contrast, the larvae reared on a natural diet (N = 80) were more resistant to the fungus: No major morphological changes were observed on their cuticles 24 hours after the treatment termination, save for a few black spots (Fig 1). More black spots appeared 24 hours later, but to a lesser extent than observed on the larvae fed a semi-artificial diet, and mortality reached only 45%. Photographs of insects after fungal infection, reared on semi-artificial or natural diets, are presented in Fig 1.

The influence of diet on insect development and weight was also examined. Thirty-five 5DL7 larvae reared on a Sehnal diet and 23 5DL7 larvae reared on beeswax were used. Arrows indicate small black spots (place of fungal invasion), which were rarely present on the cuticle surface when larvae were fed only on natural wax. No significant differences in body weight were found between the two groups (193.2 ± 6.5 mg for the beeswax-fed larvae N = 23; 189.1 ± 4.5 mg N = 35 for the semi-artificial diet-fed larvae; p = 0.5980; F_(22,34)_ = 1.329; see also S1 Dataset)

#### Various bacterial microflora on the cuticle of both *G*. *mellonella* populations

**Fig 2:**
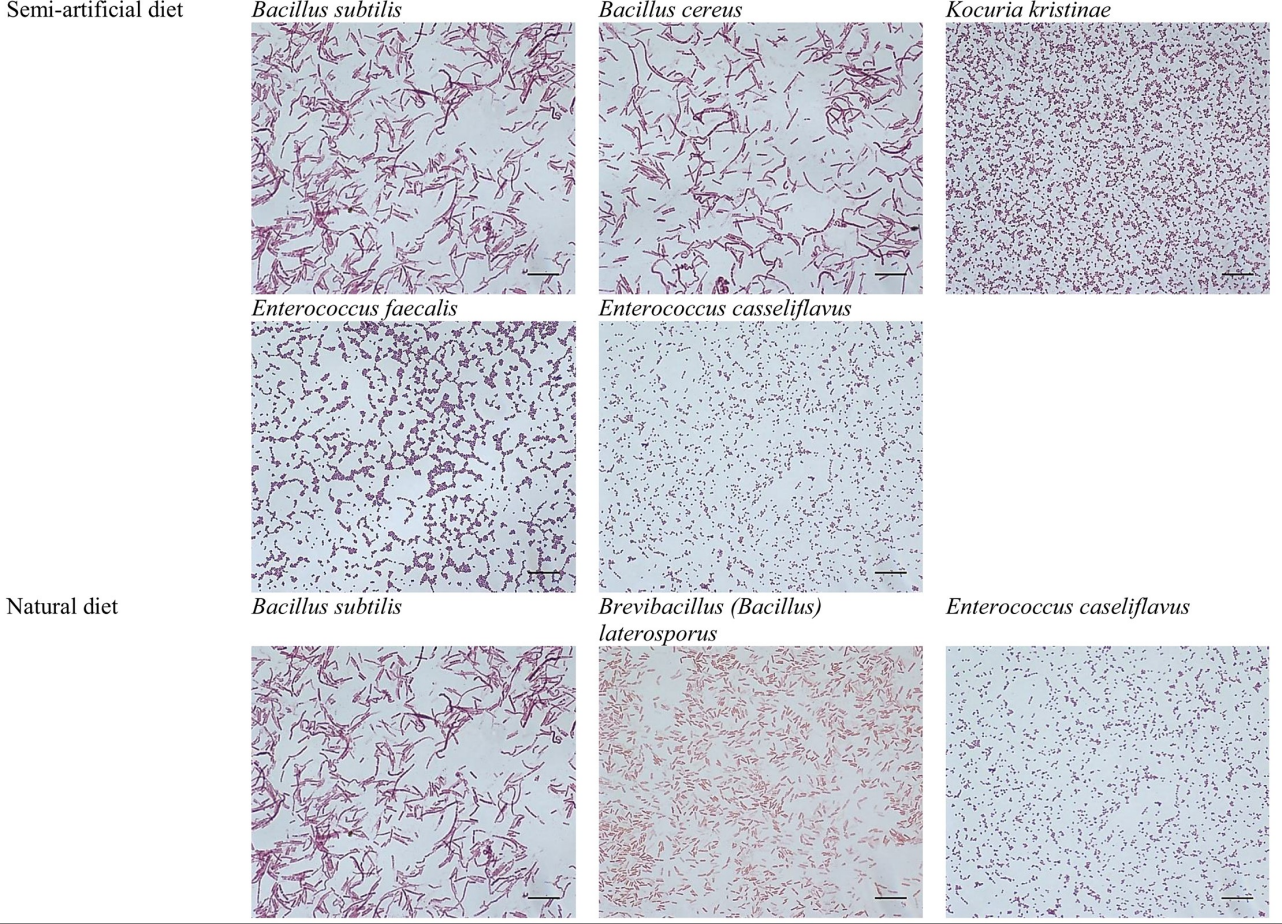
**The bacteria identified in the microbial cultures from the cuticle surface of *G*. *mellonella* larvae maintained on a semi-artificial (A) and natural (B) diets.** Gram staining was used to identify bacteria species.

Different bacterial flora were observed on the cuticle surfaces of the two groups of *G*. *mellonella* larvae (Table 1, Fig 2, and S1 Table). Five bacterial species were found on the larvae reared on the Sehnal diet: *Bacillus sublitis*, *Bacillus cereus*, *Kocuria kristinae*, *Enterococcus caseliflavus* and *Enterococcus faecalis*. However, only three species were identified on the cuticle of larvae fed on beeswax: *B*. *subtilis*, *Brevibacillus laterosporus* and *E*. *casseliflavus*. While *B*. *subtilis* and *E*.*casseliflavus* were both present on the cuticles of both groups of larvae, extensity was higher in semi-artificial fed larvae. The highest extensity was observed in the case of *B*. *laterosporus* (68%).

### GC/MS analysis of the fatty acid composition of the *G*. *mellonella* cuticle

**Fig 3:**
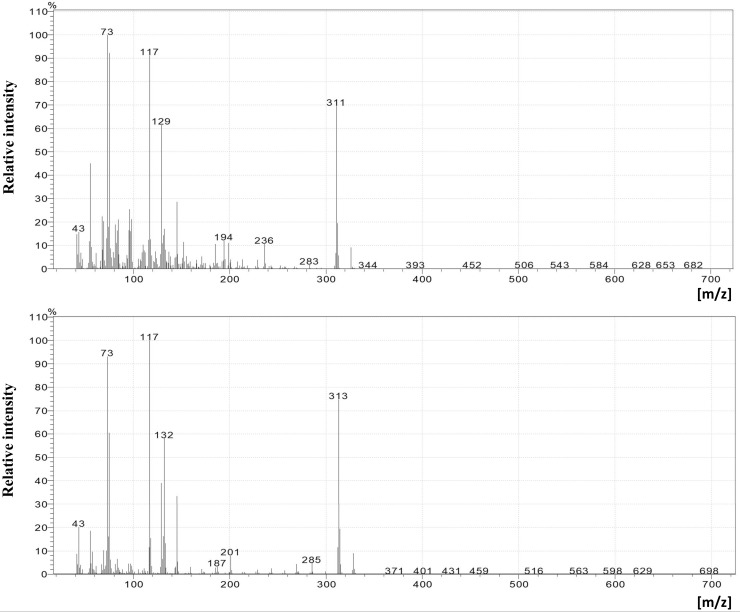
Mass spectrum of the trimethylsilyl (TMS) ester of hexadecenoic acid (A) and hexadecanoic acid (B).

**Fig 4:**
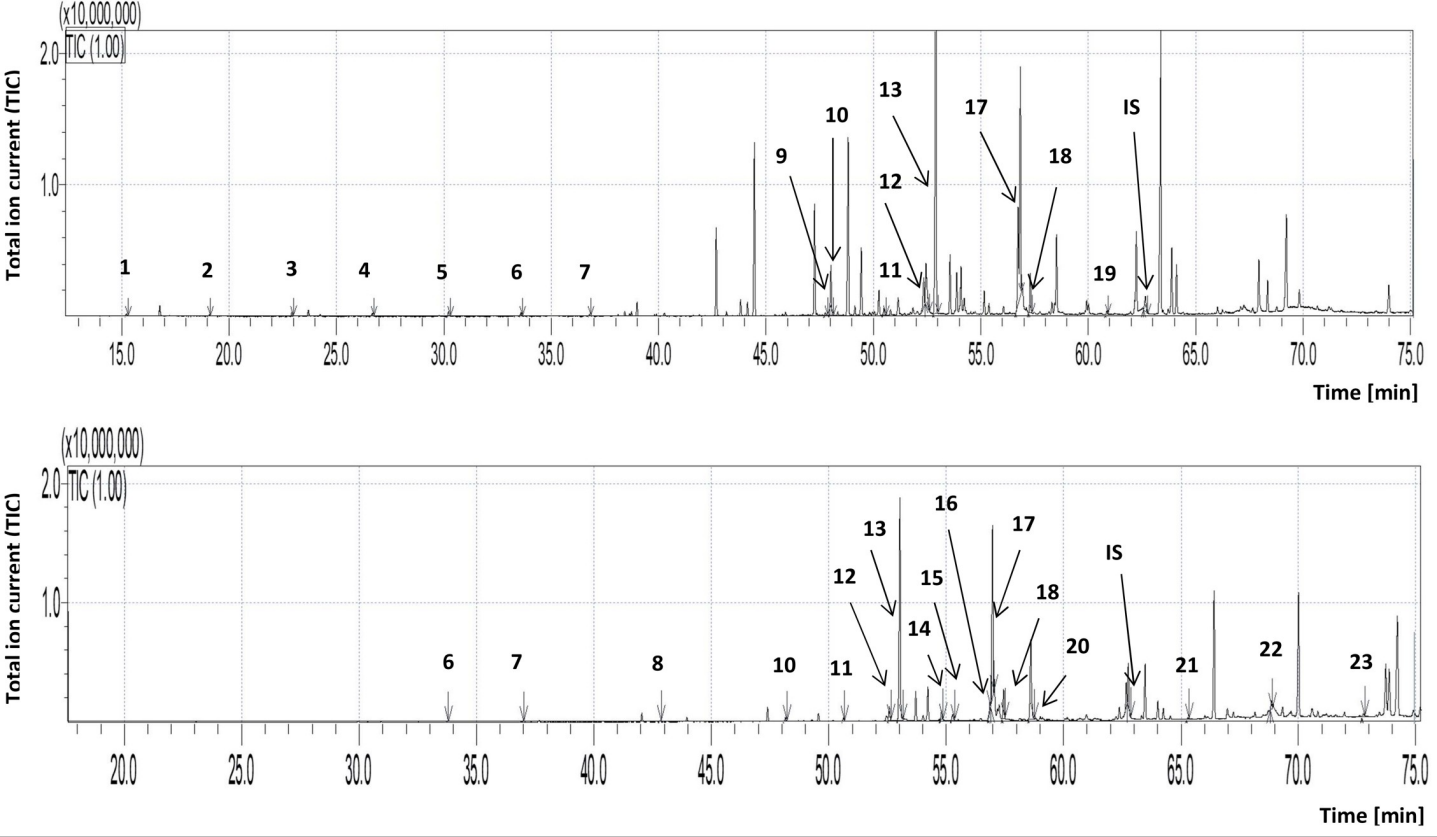
**The total ion current (TIC) of fatty acids (TMS esters) of the dichloromethane extract from *Galleria mellonella* larvae maintained on a semi-artificial (A) and natural diet (B).** IS- internal standard- 19-methylarachidic acid; fatty acids and molecular ions: **1**- butanoic acid (C4:0, m/z = 160), **2-** pentanoic acid (C5:0, m/z = 174), **3-** hexanoic acid (C6:0, m/z = 188), **4-** heptanoic acid (C7:0, m/z = 202), **5-** octanoic acid (C8:0, m/z = 216), **6-** nonanoic acid (C9:0, m/z = 230), **7-** decanoic acid (C10:0, m/z = 244), **8-** dodecanoic acid (C12:0, m/z = 272), **9-** tetradecenoic acid (C14:1, m/z = 298), **10-** tetradecanoic acid (C14:0, m/z = 300), **11-** pentadecanoic acid (C15:0, m/z = 314), **12-** hexadecenoic acid (C16:1, m/z = 326), **13-** hexadecanoic acid (C16:0, m/z = 328), **14-** heptadecenoic acid (C17:1, m/z = 340), **15-** heptadecanoic acid (C17:0, m/z = 342), **16-** octadecadienic acid (C18:2, m/z = 352), **17-** octadecenoic acid (C18:1, m/z = 354), **18-** octadecanoic acid (C18:0, m/z = 356), **19-** eicozenoic acid (C20:1, m/z = 382), **20-** docozanoic acid (C22:0, m/z = 412), 21- heneicosenoic acid (C21:1, m/z = 396), **22-** tetracosanoic acid (C24:0, m/z = 440), **23-** hexacosanoic acid (C26:0, m/z = 468).

The results of the cuticular lipid extraction varied according to the diet of *G*. *mellonella* (Table 2; see also S2 Dataset). The analysis included 20 larvae reared on the Sehnal diet and 30 larvae reared on beeswax. Examples of the mass spectra of the trimethylsilyl (TMS) esters of hexadecanoic acid (C 16:0) and hexadecenoic acid (C 16:1) are given in Fig 3, and the total ion current (TIC) of the trimethylsilyl esters (TMS) of FFAs (after derivatization) extracted by dichloromethane from both groups of *G*. *mellonella* larvae are presented in Fig 4.

**Table 2:** Quantitative summary of the experiment: Numbers of used differently fed *Galleria mellonella* larvae and masses of extracts.

Type of diet	Number of insects	Masses of insects (g)	(mg) of extract per (g) of insect
**Semi-artificial**	20	3.56	0.28
**Natural**	30	4.89	0.20

The qualitative and quantitative GC-MS analyses found that the identified FFAs contained between four and 26 carbon atoms in the alkyl chain. The two groups of larvae demonstrated different profiles of FFAs. Short-chain FFAs, such as C4:0, C5:0, C6:0, C7:0 and C8:0, were observed only in larvae reared on semi-artificial Sehnal diet, while more long-chain FFAs ranging from C21:1 to C26 were found in the beeswax-fed larvae. Table 3 lists all the fatty acids identified in extracts obtained from the insects, calculated as μg/g insect body (see also S3 Dataset).

**Table 3:** List of the free fatty acids (FFAs) extracted from the cuticle of *G*. *mellonella* larvae maintained on the semi-artificial and natural diet. All results were calculated as μg/g insect body.

**FFA**	**Semi-artificial diet** **(mean μg/g ± SD)**	**Statistical significance**	**Natural diet** **(mean μg/g ± SD)**	**Semi-artificial diet** **%** **(the percentage composition)**	**Natural diet** **%** **(the percentage composition)**
**C 4:0**	0.72 ± 0.28	*	nd	0.15	nd
**C 5:0**	0.36 ± 0.13	**	nd	0.07	nd
**C 6:0**	1.10 ± 0.48	*	nd	0.23	nd
**C 7:0**	0.95 ± 0.34	**	nd	0.19	nd
**C 8:0**	1.63 ± 0.06	***	nd	0.34	nd
**C 9:0**	1.34 ± 0.17	***	0.05 ± 0.004	0.28	0.07
**C 10:0**	0.96 ± 0.30	**	0.04 ± 0.01	0.19	0.05
**C 12:0**	nd	***	0.11 ± 0.02	nd	0.14
**C 14:1**	2.02 ± 1.35		nd	0.41	nd
**C 14:0**	28.69 ± 9.39	**	0.44 ± 0.02	5.89	0.58
**C 15:0**	7.12 ± 5.79		0.36 ± 0.02	1.46	0.47
**C 16:1**	18.34 ± 13.72		1.51 ± 0.07	3.77	2.00
**C 16:0**	329.52 ± 154.13	*	30.25 ± 0.14	67.73	39.86
**C 17:1**	nd	*	0.38 ± 0.18	nd	0.50
**C 17:0**	nd	***	1.47 ± 0.13	nd	1.93
**C 18:2**	nd		1.29 ± 1.14	nd	1.71
**C 18:1**	80.19 ± 49.58		23.25 ± 2.99	16.48	30.64
**C 18:0**	12.27 ± 2.72	**	3.68 ± 0.05	2.52	4.85
**C 20:1**	1.33 ± 0.37	**	nd	0.27	nd
**C 21:1**	nd	***	11.82 ± 0.28	nd	15.60
**C 22:0**	nd	**	0.13 ± 0.03	nd	0.17
**C 24:0**	nd	***	0.76 ± 0.08	nd	1.00
**C 26:0**	nd	***	0.36 ± 0.03	nd	0.48
**Sum**	**486.54**		**75.90**	**100**	**100**

nd–not detect

*p< 0.05

**p< 0.001

***p< 0.0001

Fifteen fatty acids were found on *G*. *mellonella* larvae reared on the semi-artificial diet, and sixteen on those reared on the natural diet. Eight fatty acids were found in both *G*. *mellonella* profiles: C9:0, C10:0, C14:0, C15:0, C16:1, C16:0, C18:1 and C18:0. Seven FFAs detected on larvae reared on the semi-artificial diet were absent in larvae reared on natural beeswax: C4:0, C5:0, C6:0, C7:0, C8:0, C14:1 and C20:1. Eight fatty acids were present on the cuticle of the larvae reared on the natural diet but were absent on those reared on the semi-artificial diet: C12:0, C17:1, C17:0, C18:2, C21:1, C22:0, C24:0 and C26:0. In both profiles, C16:0 and C18:1 predominated.

In addition, it was observed that the FFAs on the cuticle of the beeswax-fed larvae were present in lower amounts than the insects fed a semi-artificial diet: these total amounts being 486.54 μg/g for the larvae reared on the Sehnal diet and 75.9 μg/g for those reared on beeswax, i.e. six-times lower.

Statistically significant differences were observed between the two groups with regard to the amounts of FFAs on their cuticles (Table 3). It was found that the larvae reared on beeswax demonstrated 65-times less tetradecanoic acid (C14:0), 12-times less hexadecenoic acid (C16:1), 11-times less hexadecanoic acid (C16:0), and four-times less octadecenoic (C18:1) and octadecanoic acids (C18:0) than the larvae reared on the Sehnal diet. However, comparing the percentage shares of each individual FFA in the pool of all extracted FFAs in both groups of insects, the differences between them are no longer so marked. For example, the concentration of hexadecanoic acid (C16:0) was 11-times lower in the cuticles of beeswax fed larvae than those fed Sehnal diet, if calculated as μg/g insect body; however, the value is only two-times lower when presented as percentage share in the pool of all extracted FFAs. Similarly, the level of octadecenoic acid (C18:1) was four-times lower in naturally-fed larvae when measured as μg/g insect body, but only 1.8 times lower when measured as percentage share (Table 3).

The one-way ANOVA and Tukey’s *post hoc* test were used to evaluate the statistical significance of differences between the amounts of the identified FFAs. The tests were performed separately within each group of larvae. In the larvae fed the Sehnal diet, the total amount (μg/g insect body) of hexadecanoic acid (C16:0) was significantly higher (p<0.0005) than that of the rest of the free fatty acids in this group. Among the larvae fed beeswax, the total amounts (μg/g insect body) of hexadecanoic acid (C16:0), octadecenoic acid (C18:1), octadecanoic acid (C18:0) and heneicosenoic acid (C21:1) were significantly higher (p-values ranging from p<0.05 to p<0.0001) than those of the rest of the free fatty acids.

## Discussion

The present work clearly shows that differences exist in the cuticle components of *G*. *mellonella* larvae reared on various diets. It is important to emphasize that no differences in appearance, behavior, duration of development and fecundity were seen between the two groups of insects. No differences in the weights of 5DL7 larvae were observed either. Both the larvae fed on beeswax and those reared on the Sehnal diet were similar in appearance and were in good condition, indicating that both diets were energetically equivalent.

This work confirms previous findings that the most important and predominant cuticular FFAs in greater wax moth larvae are heneicosanoic (C16:0) and octadecenoic (C18:1) acids [12, 20]. One particularly interesting observation is that the third most abundant FFA is tetradecanoic acid (C14:0) among the larvae kept on a semi-artificial diet, but heneicosenoic acid (C21:1) among the larvae reared on beeswax. Heneicosenoic acid is not a very common acid in nature, and its biological role is unclear; however, we assume that it may play a protective role in larvae during fungal invasion. If so, it is possible that FFAs can play an essential role during fungal infection and changes in their composition may be responsible for changes in sensitivity to *C*. *coronatus* fungus. The cuticles of larvae fed beeswax contained more long-chain FFAs, such as C21:1, C22:0, C24:0, and C26:0, while those on a semi-artificial diet contained more short-chain fatty acids: C4:0, C5:0, C6:0, C7:0, and C8:0.

In addition, differences were observed between the two groups of larvae regarding the concentrations of free fatty acids. More specifically, FFA concentrations were lower in insects reared on natural wax than those on the semi-artificial diet: 65x less tetradecanoic acid (C14:0), 12x less hexadecenoic acid (C16:1), 11x less hexadecanoic acid (C16:0), and 4x less octadecenoic (C18:1) and octadecanoic acids (C18:0). Boguś et al (2010) report that C16:0, C16:1, C18:0, C18:1, C18:2, C18:3, C20:0 and C20:1 inhibited the growth of *C*. *coronatus*, but C16:1 greatly increased its virulence. In the present study, the level of hexadecenoic acid was found to be 12x higher in larvae reared on a semi-artificial diet, and this may be responsible for the higher sensitivity of these insects to fungal infections. Moreover, Boguś et al (2010) note that low concentrations of C5:0, C6:0, C6:2 and C7:0 enhanced sporulation of *C*. *coronatus*. In the present study, three of these short-chain free fatty acids (C5:0, C6:0 and C7:0) were observed on the cuticle of larvae raised on the Sehnal diet, but not on that of the beeswax-fed larvae. Smith and Grula demonstrate that short-chain saturated fatty acids extracted from *Heliotis zea* larvae can inhibit the growth of *Beaveria bassiana* [21], while other studies report that some of the fatty acids taken from insect cuticle have a toxic effect against *B*. *bassiana*, *Paeciliomyces fumoroseus* and *Conidiobolus obscurus* [22–24]; e.g. Saito and Aoki showed that the short-chain fatty acids (from C6:0 to C12:0) inhibited the conidial germination and also hyphal growths of *B*. *bassiana* and *P*. *fumosoroseus*; on the other hand, some long-chain fatty acids (C14:0, C16:0, C18:0 and C18:1) supported the growth of both fungi. It therefore seems that the high susceptibility of larvae fed a semi-artificial diet to *C*. *coronatus* infection observed in the present study might result from the presence of significantly higher concentrations of C16:1 on their cuticles, as well as the presence of C5:0, C6:0 and C7:0, which are absent from the beeswax-fed larvae resistant to *C*. *coronatus*.

We propose that these differences in cuticular FFA profiles could be attributed to differences in diet. Beeswax is a mixture of more than 300 compounds including hydrocarbons, free fatty acids, fatty alcohols and esters of fatty acids [25, 26]. It is also important to note that beeswax can vary among families and breeds, and that wax production may also be dependent on the genetics of the bees [27]. It has also been shown that while beeswax has antimicrobial activity, it can also contain certain microbes [26, 28]: the bacteria and yeasts found in the honeycomb are mostly derived from the bees. Sackelt and colleagues found that some species of *Bacillus*, *Micrococcus* and *Saccharomyces* could be readily isolated from honeycombs, as well as from bees [29]. Other studies have found the intestinal microbiota of bees to comprise 1% yeasts, 27% Gram-positive bacteria like *Bacillus*, *Streptococcus* and *Clostridium* spp, and 70% Gram-negative or variable bacteria, e.g. *Citrobacter*, *Enterobacter*, *Escherichia coli*, *Flavobacterium*, *Klebsiella* and *Pseudomonas* [30]. However, most of the microbes found in honey remain dormant, thus demonstrating the antibacterial activity of honey [28].

Banville et al. observed that larvae deprived of nutrition demonstrated increased susceptibility to *Candida albicans* infection, as well as a slight reduction in hemocyte density; however, the hemocytes from the starved larvae were as effective at killing fungal cells as those from the well-fed ones [31]. They concluded that food deprivation can reduce the cellular and immune responses of the greater wax moth and increase its susceptibility to infection. Li and coworkers showed that calorie restriction reduces the expression of arylophorin and lipoprotein in *Bombyx mori*, and that lack of food can adversely affect metabolism and lead to a decreased immune response [32]. Our present findings show that the type of food consumed by *G*. *mellonella* larvae can be of particular significance during infection by *C*. *coronatus*, as larvae reared on beeswax were less sensitive to the pathogen.

Insects are very often colonized by microorganisms. Some of them are pathogenic but some part of these microorganism cane be beneficial or even needed by the insect host. The insect cuticle (also known as the exoskeleton) acts as a vital important physical barrier against a number of microbes and can also serve as a substrate for many others [33]. Microorganisms can support their insect hosts against various invasion of pathogens by multiple mechanisms, e.g. competition for nutrients or space, production of toxins and activation of the insect immune response. Our findings highlight the variations in the bacterial composition of the cuticle associated with the diet of the insect. The bacteria *B*. *subtilis* and *E*. *casseliflavus* were observed in both groups of larvae, while *B*. *cereus*, *E*. *faecalis* and *K*. *kristinae* were identified on the cuticles of larvae reared on semi-artificial food. All of these bacteria are opportunistic and have been implicated in many diseases in human populations [34–36]. These three bacteria (*B*. *cereus*, *E*. *faecalis* and *K*. *kristinae*) may have been transferred to the larvae cuticle from food and/or the surface of the human skin: Sehnal food is prepared in the laboratory from everyday groceries like corn and wheat flour, bran and milk powder.

*B*. *laterosporus* were observed only in larvae fed on beeswax. It is worth emphasizing the fact that *B*. *laterosporus* shows insecticidal activity. Orlova and colleagues note that crystal-forming strains of *B*. *laterosporus* (921 and 615) possessed insecticidal activity against larvae of the mosquitoes *Aedes aegypti*, *Anopheles stephensi* and *Culex pipiens* [37]. The bacterium *B*. *laterosporus* has also shown to be insecticidal against various insects (different orders) including *Anthonomus grandis*, *Anticarsia gemmatalis*, *Tenebrio molitor*, *Spodoptera frugiperda* [38–40], as well as the common housefly *Musca domestica* [41, 42]. On the other hand, different *B*. *laterosporus* strains show activity against a broad spectrum of microbes including many bacteria and fungi [43, 44]. This antifungal potential can explain the higher resistance of *G*. *mellonella* reared on beeswax to *C*. *coronatus* infection. In addition, *Brevibacillus* species have been accredited with the production of a wide range of enzymes, antibiotics and probiotics [43]. Hence, the bacterial composition of the cuticle may play a role in preventing infection by *C*. *coronatus* fungus.

We believe that these differences are closely connected with the type of insect diet. Unfortunately, little information exists about insect nutrition and its role in lipid metabolism, particularly regarding the synthesis, transport and distribution of cuticular free fatty acids. Unfortunately, we cannot account for the significantly higher total FFA content (6.4 times higher) on the cuticle of larvae fed a semi-artificial diet than on larvae fed beeswax.

In summary, our findings demonstrate a correlation between the type of food (different source of nutrients) and the sensitivity of *G*. *mellonella* larvae to fungal infection. Larvae reared on natural beeswax demonstrate a distinctive profile of free fatty acids and bacterial species on the cuticle surface, which probably leads to significantly greater ability to curtail or eliminate the pathogen.

Knowledge of the interactions between entomopathogenic fungi and insects is extremely valuable for two key reasons: firstly because these fungi can be used as bioinsecticides for crop protection and food preservation, and secondly, because *G*. *mellonella* can be a great model to study immunology processes and pathogen resistance, which can be also useful for human model diseases. It is therefore important to understand the mechanism of infection and the influence of the invading fungus on insect immunology, as this may provide an insight into how insects defend themselves against entomopathogenic fungi. Moreover, our results confirm that the type of diet consumed by the insect can influence its resistance, and provide important information regarding why different species are more or less resistant to pathogens. In addition our findings shed further light on the defenses used by insects against fungal invasion. This knowledge can be valuable for both basic research and for practical applications, as it represents a valuable tool for understanding the complex interactions between insects and entomopathogenic fungi.

## Supporting information

S1 Table The occurrence of bacterial species on the cuticle of *G*. *mellonella* larvae. (XLSX)

S1 Dataset Body weights of *G*. *mellonella* larvae reared on semi-artificial and natural diet-raw data. (XLSX)

S2 Dataset Numbers of used differently fed wax moth larvae and masses of extracts-raw data. (XLSX)

S3 Dataset The free fatty acid (FFAs) contents extracted from the cuticle of the *G*. *mellonella* larvae—Raw data. (XLSX)
